# Ultrasound propagation speed and mass changes by application of different accelerated artificial ageing tests in granitic materials

**DOI:** 10.1038/s41598-026-56811-z

**Published:** 2026-06-22

**Authors:** J. García-Talegón, A. C. Iñigo, J. Paredes, R. A. Sepúlveda Correa, L. Martín Barajas, E. Azofra Agustín

**Affiliations:** 1 Departamento de Geología, Universidad de Salamanca, Salamanca, Spain; 2 Instituto de Recursos Naturales y Agrobiología de Salamanca (IRNASA-CSIC), c) Cordel de Merinas 40‐52, 37008 Salamanca, Spain; 3 Departamento de Estadística, Universidad de Salamanca, Salamanca, Spain; 4 Departamento de Historia del Arte, Universidad de Salamanca, Salamanca, Spain

**Keywords:** Weathering, Freezing/thawing, Salt crystallisation, Ultrasound propagation speed, Weight loss/gain, Three-way mixed MANOVA, Engineering, Materials science

## Abstract

In this work the ultrasound propagation speed on the three spatial planes (Vx,Vy,Vz) and the weight loss/gain of three ornamental granites (Ávila, Spain) were determined before, during, and after being subjected to 90 cycles of three types of accelerated ageing processes (typical of cold regions): a) freezing/thawing and cooling/heating, b) salt crystallisation, and c) freezing/thawing and cooling/heating + salt crystallisation. A three-way mixed multivariate analysis of variance (MANOVA) was used for data analysis. Significant variations in the ultrasonic propagation speed and weight loss/gain of the different granites were observed for the three types of accelerated artificial ageing processes compared to the data obtained from the quarry samples, with the Airón Biotitic Granite (G) variety being the most altered granite and the combined freezing/thawing and cooling/heating + salt crystallisation accelerated artificial ageing test being the most damaging. To determine the durability of the different varieties of granite against the accelerated artificial ageing tests studied, the estimated data of compressive strengh, obtained from the ultrasound propagation speed data, were determined, with the Ávila Grey Granite (C) being the most resistant to the ageing tests studied.

## Introduction

The area surrounding Ávila (Spain) has a Mediterranean climate with a strong continental influence. It is a cold region, characterised by abrupt thermal oscillations, with differences of up to 31 °C between day and night. These conditions favour thermoclastic processes that induce differential thermal expansion among the mineral grains in granitic materials. The resulting stresses generate micro-discontinuities that facilitate the circulation of fluids (water, dissolved salts, etc.)^1,2^. In humid regions with low temperatures, rock weathering by freezing (gelifraction) also constitutes a significant degradation mechanism. The freezing of water produces a volumetric expansion of approximately 9% of the original volume, leading to stress concentration and tensile damage in the micropores^3,4^. When rocks thaw, water infiltrates through the newly formed voids, causing further microcracking^2,5–12^.

In addition, under conditions of permanent humidity, weathering is also produced by precipitation and crystallisation of salts within the pore network^13–19^. Dissolution and precipitation reactions occur as a result of fluid–rock interactions at the interface^20^. Consequently, the most frequent climate-driven degradation mechanisms affecting granitic building stones are as follows^7,21–24^: a) freezing/thawing and cooling/heating; b) salt crystallisation; and c) the combined effect of freezing/thawing and cooling/heating + salt crystallisation.

These ageing processes can be recreated under controlled conditions in climatic simulation chambers, which allow the reproduction of the deterioration phenomena observed in many historical monuments in Ávila, (Spain, a 1985 UNESCO World Heritage Site). In this regard, our research group has also conducted in situ assessments of stone weathering forms in Villamayor sandstone (Salamanca, Spain) using terrestrial laser scanner intensity data^25^.

Several researchers have examined changes in the physical and mechanical properties of rocks through laboratory-induced degradation processes ^9,11,21,22,26–29^. Liu et al.^30^ analysed rocks subjected to freeze/thaw cycles using both physical tests (ultrasonic propagation speed, mass variations, porosity, etc.) and mechanical tests (compressive, tensile, and impact resistance, modulus of elasticity, etc.).

Alonso et al.^31^ applied the UNE-EN 12370 standard to assess the durability of four commercial granites from Galicia under degradation caused by salt crystallisation. According to this standard, to evaluate the alteration produced by this type of ageing it is sufficient to determine the variations in ultrasonic propagation speed and the variations in mass between the initial and final states. Although this standard is not formally applicable to crystalline rocks with porosity below 5 %, the authors argued that it can still yield valuable information on potential damage and long-term behaviour of low-porosity materials. Mass losses were negligible (<0.04 %) and mainly attributed to the detachment of biotite grains. After testing, all of the granites analysed exhibited a decrease in ultrasound propagation speed indicating the formation of new microcracks the widening of pre-existing ones.

An important variable to be controlled in both artificial ageing and the application of conservation treatments is the measurement of the ultrasound propagation speed (non-destructive), which could indicate a decrease in the cohesion of the minerals as a result of the application of T1, T2, and T3 ageing treatments^5,8,32–36^ and, as a consequence, a decrease in their compressive strengh^21^, or, conversely, an increase in this parameter due to application of a conservation treatment (consolidation)^36,37^.

Most studies in this field focus on observing how a specific type of ageing affects different kinds of rock substrates. Thus, the following cases can be mentioned: a) thermal shock ^2,38^, b) freezing/thawing ^3–6,8–12,27,28,30,33–36,39^, c) salt crystallisation^14–19,31,40–44^. In these latter cases, it is not possible to establish a logical relationship between the degradation produced by several ageing processes; however, it is possible to analyse the effects of one of them on specific properties or intrinsic characteristics of each of the stone materials studied. Nevertheless, we find several other works that, within the same study, include the response of different stone materials to various ageing processes (thermal shock, freezing/thawing, or salt crystallisation)^22,24^, (thermal shock or freezing/thawing)^1,26,29^. In this case, ageing methods are applied individually, following a specific standard. These latter works do allow for the correlation of damage produced by different ageing processes across a range of stone materials.

In some of our previous studies^7,45,46^, we examined the responses of different stone materials to ageing caused by salt crystallisation (sulphates). In these cases, the damage produced was much greater than that caused by freezing/thawing ageing, since sodium sulphate undergoes a greater volumetric increase upon crystallisation than water does when it turns into ice, thereby leading to a more intense wedge effect. At other times, we combined several types of ageing, applying new methodologies (freezing/thawing + heating/cooling^3,7,^^21,36,45,46^, and freezing/thawing + heating/cooling + salt crystallisation^7,21,45,46^). In this way, we were able to increase the degree of deterioration compared to ageing processes applied separately, and we reproduced a wider range of weathering forms that could not have been achieved without such combinations. This approach was undertaken because we consider that the forms of weathering observed in monuments are never the result of a single process instead, two or more processes are always involved. Thus, this approach brings us closer to reality. That said, it is true that the presence of certain weathering forms is primarily associated with a single process that prevails over other possible processes, which play a minor role, due to the specific microenvironment developed for their occurrence, whether inside or outside the monuments.

The main objective of this work was to investigate the influence of freezing/thawing cycles, with or without the presence of salt crystallisation (sulphates), on the weight loss/gainand ultrasound propagation speed on the three spatial axes (Vx,Vy,Vz). A three-way mixed multivariate analysis of variance (MANOVA) was used to analyse the variations in these variables for three of the most widely used granites in the construction and subsequent interventions of historical monuments in Ávila (Spain), before, during, and after subjecting them to 90 cycles of the three types of artificial ageing tests used. In addition, the regression model proposed by Iñigo et al.^21^ was used to analyse the data on the ultrasonic propagation speed in the studied granites, in order to estimate the compressive strengh to determine their durability against the studied ageing tests.

## Materials

For the construction of religious monuments during the Romanesque period (12^th^–13^th^ centuries) in the city of Ávila (Spain, a 1985 UNESCO World Heritage Site), Silicified Granite (as a Global Heritage Stone Resource) ^23^—used in its varieties known as Bleeding Stone and Ochre Granite, varieties traditionally known as *Alterite* (locally applied to palaeoweathered, highly silicified granites, are found in the intermediate and upper part of a complex palaeoweathering mantle/profile developed on the Iberian Hercynian Basement formed through an opal-CT silicification process, kaolinization, remobilization of iron oxyhydroxides,and later processes of hydromorphy)—was employed, together with Ávila Grey Granite in the plinths of these monuments. Silicified granites continued to be used until the 18^th^ century, although only in the vaults of certain palaces and religious monuments and in decorative elements^23^. One possible explanation is the depletion of the original quarries; however, in the small nearby village of La Colilla (7 km from city of Avila), a historical quarry where Silicified Granite was extracted has been preserved and protected, and it is currently used exclusively for the restoration of Ávila’s monuments.

**Fig. 1:**
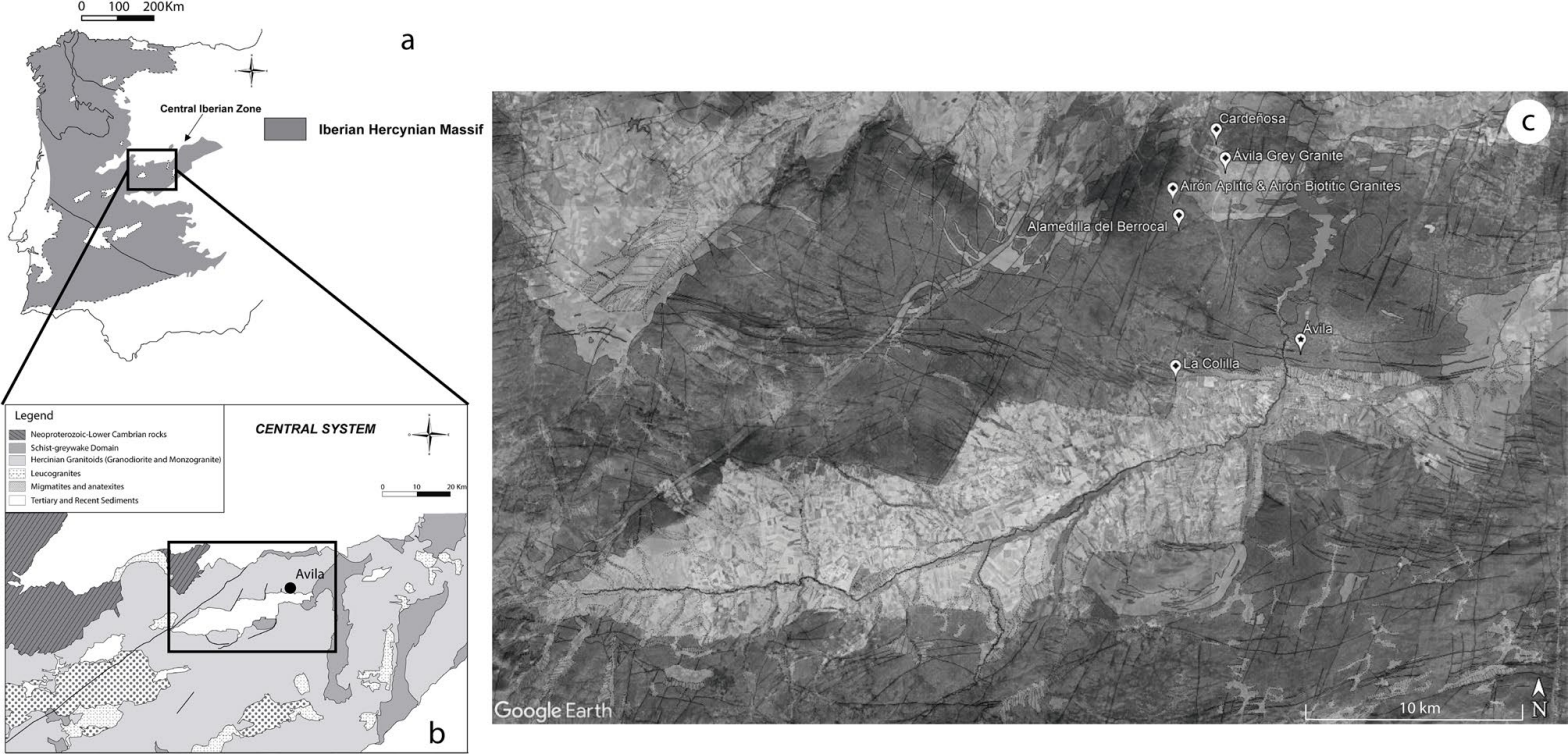
Geological settings of the studied area. (**a**) Location of the Central System within the Central Iberian Zone of the Iberian Hercynian Massif (simplified from Julivert et al. 1975). (**b**) Regional geological map of the Central System highlighting the studied sector around Ávila (modified from GEODE, Continuous Digital Geological Map of Spain, accessed May 21, 2026). (**c**) Satellite view showing the precise locations of the studied Ávila Granite quarries, including Cardeñosa, Ávila Grey Granite, Alamedilla del Berrocal, Airón Aplitic & Airón Biotitic Granites, and La Colilla (Google Earth imagery, September 10, 2025).

**Fig. 2:**
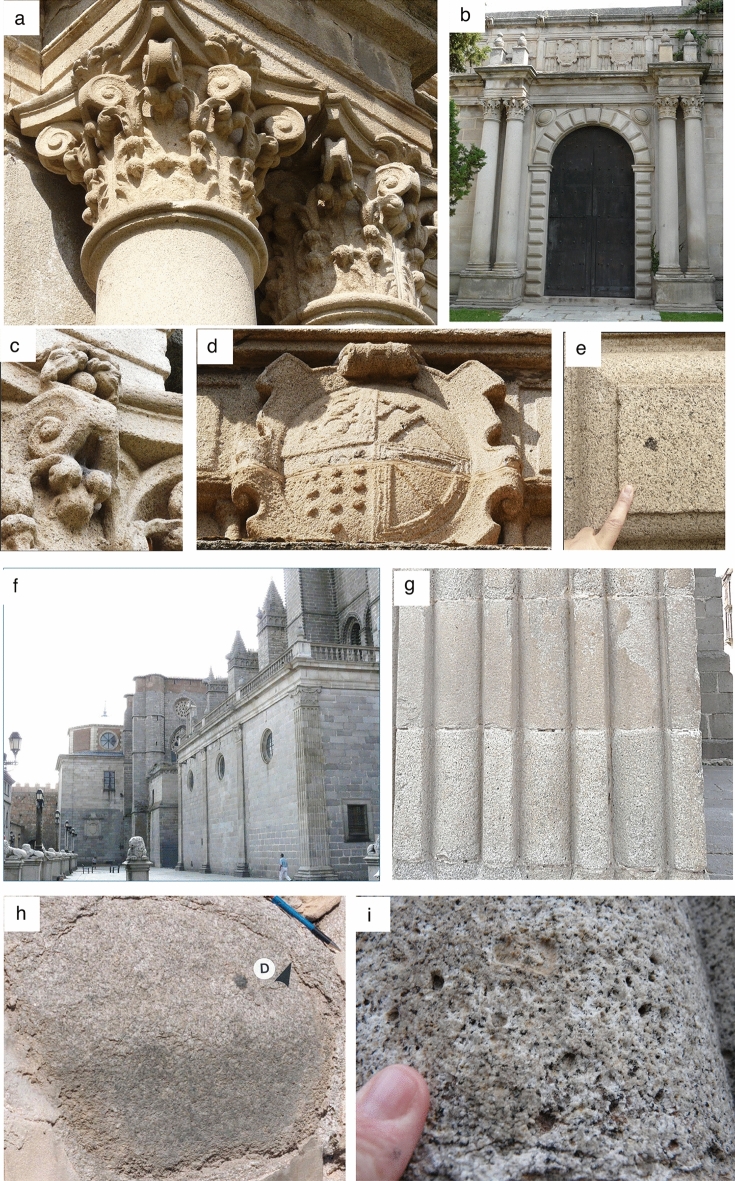
Location of Ávila Grey Granite (C), Airón Aplitic Granite (F), and Airón Biotitic Granite (G) in the southern portal of the Mosén Rubí Chapel (16th c.) and in the northern chapels of Ávila Cathedral (16th–18th c.): (**a**) Slightly altered C capitals exhibiting a light beige patina. (**b**) Southern portal of the Mosén Rubí Chapel: attached G columns, F capitals, and F heraldic shields. (**c**) Detail of an F capital with a beige patina. (**d**) Detail of an F heraldic shield showing a beige patina. (**e**) Detail of the G door jamb. (**f**) View of the northern chapels of Ávila Cathedral. (**g**) Chromatic alternation of G (showing scaling) and F. (**h**) Scaling in C. (**i**) Pitting-type deterioration affecting G.

In the present study, we focus on the granites *s.s.* (lower part of palaeoweathering profile) used in Ávila from the 13^th^ to the 18^th^ century, namely the medium- to coarse-grained Ávila Grey Granite (granite from Cardeñosa) (C) and the fine grained granites (Airón Aplitic Granite (F) and Airón Biotitic Granite (G) from Alamedilla of Berrocal (Figs. 1 and 2a-i) from the 16^th^ to the 18^th^ century. In wall construction, medium- to coarse-grained slightly porphyritic Ávila Grey Granite is the predominant material (Fig. 2f, h), whereas fine grained granites are preferentially used in the main façades of religious monuments, palaces, and convents, particularly in architectural and decorative elements such as columns, capitals (Fig. 2a, c), lintels, balustrades, and coats of arms (Fig. 2d), (e.g. Chapel of Mosén Rubí´s Chapel, 16^th^ c. in Avila city, Fig. 2a-e). In addition, the chromatic alternating pattern of these granites can be observed in the exterior walls of the chapels located on the northern and eastern sides of the Avila Cathedral (Fig. 2g-i), (e.g. Chapel of Saint Segundo, Avila city 18^th^ c.). The extensive use of fine grained granites (Aplitic and Biotitic varieties) is attributed to their capacity to yield large blocks during extraction, which is especially advantageous for producing monumental columns (Fig. 2b), such as those on the south portal (Fig. 2b) of Mosén Rubí´s Chapel^47^.

The quarrying area of the granites analysed in this work forms part of the Variscan plutonic suite of the Cardeñosa–Ávila sector, within the Iberian Massif. This region is assigned to the Western Domain of the Central System (Central Iberian Zone) and is characterised by variable metamorphic grades and an extensive development of granitoid intrusions (Peraluminous granodiorites and monzogranites) (Fig. 1).

**Fig. 3:**
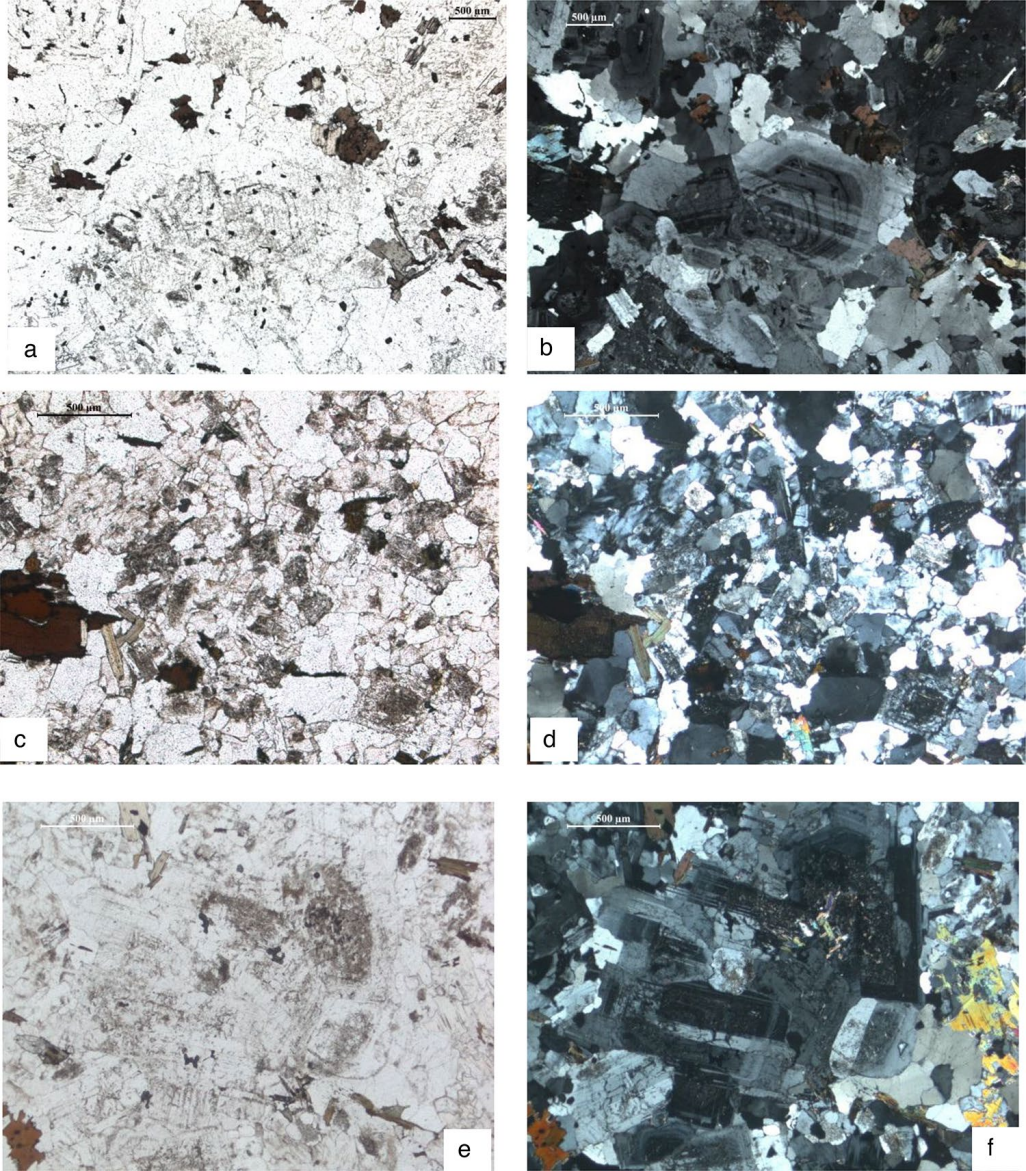
Petrographic characterisation using thin section photomicrographs of the Ávila Grey Granite (**a** and **b**), Airón Aplitic Granite (**c** and, **d**) and Airón Biotitic Granite (**e** and **f**). (a and b, scale bar = 500 µm) Plane and cross-polarised light of zoned plagioclase crystals and the biotite crystals are slightly chloritised. (c and d, scale bar = 500 µm) Plane and cross-polarised light of the biotite crystals with sphene and zoned plagioclase crystals showing altered cores. (e and f, scale bar = 500 µm) Plane and cross-polarised light of aggregates of plagioclase crystals with altered cores and muscovite.

The Ávila Grey Granite (Cardeñosa, C) is a medium- to coarse-grained, grey (Fig. 2h), has been quarried in the village of Cardeñosa (Fig. 1), slightly porphyritic granite composed mainly of quartz, potassium feldspar, plagioclase and biotite. Accessory minerals include cordierite (commonly altered to muscovite), apatite, muscovite and zircon, while muscovite, chlorite, sphene and epidote occur as secondary minerals. Quartz displays undulatory extinction, indicating incipient recrystallisation. Potassium feldspar shows poikilitic and perthitic textures, and plagioclase is subidiomorphic and zoned, with anorthite content increasing from albitic rims to oligoclase cores (Fig. 3a-b).

Airón Aplitic Granite (F) has been quarried in the village of Alamedilla del Berrocal (Fig. 1), is a leucocratic granite with an aplitic tendency, equigranular, with fine grain. In general, the rock presents a beige colour of alteration recorded by plagioclase and iron oxides and a white tone in the potassium feldspar cystals, which stand out slightly (Fig. 2a-e); it is composed of quartz, potassium feldspar, plagioclase and biotite, as accessories are muscovite, apatite and zircon (Fig. 3c-d). Exhibiting as secondary minerals are chlorite, sphene, muscovite, opaques, iron oxides and sericite (Fig. 3c-d). Potassium feldspar has an anhedral tendency. The plagioclase is of the acid albite-oligoclase type and is partially zoned, with contents of An_17_ in the core and An_0,9_ at the edge of the crystal.

Airón Biotitic Granite (G) has been quarried in the village of Alamedilla del Berrocal, (Fig.1), is a fine-grained leucocratic granite (Fig. 2a-e). It is composed of potassium feldspar, quartz, plagioclase, biotite and/or muscovite. Abundant accessories are apatite and zircon, along with cordierite. As secondary, there is chlorite, sphene, ilmenite, muscovite, sericite (Fig. 3e-f). Aggregates of muscovite are observed as essential and belong to a tardimagmatic stage.

Based on the major‐element chemical analyses, and excluding hydrated mineral phases, the three granite varieties were classified using the CIPW normative calculation. Petrographic microscope observations are fully consistent with this chemical classification. The Q′–ANOR′ diagram indicates that the monzogranite corresponds to the most calcic variety (Ávila Grey Granite, Cardeñosa, C), whereas the other two varieties (Airón Aplitic Granite, F; Airón Biotitic Granite, G) plot within the syenogranite field and are characterised by a higher aluminous content.

Ávila Grey Granite (C) exhibits the highest plagioclase content (47.2%), Airón Aplitic Granite (F) the lowest (31.3%), and Airón Biotitic Granite (G) an intermediate value (40.5%). Quartz contents range from a minimum in granite C (28.9%) to a maximum in granite F (38.5%). Potassium feldspar is most abundant in granite F and least abundant in granite C, whereas biotite reaches its highest proportion in granite C (17.5%) and its lowest in granite G (5.7%). Conversely, muscovite shows its lowest content in variety C (0.4%).

## Methods

A sufficient amount of material was sampled to perform: (i) major‐element chemical analyses; (ii) preparation of thin sections for petrographic study; (iii) collection of a sufficient volume to produce the cubic specimens required for hydric and mechanical characterisation and ultrasonic velocity measurements of the three selected varieties; and (iv) preparation of samples to be subjected to the three accelerated artificial ageing tests.

Major‐element chemical analyses were performed at ACTLABS (Ancaster, Canada) using the 4LITHORESEARCH analytical package, with determinations carried out by inductively coupled plasma–optical emission spectrometry (ICP‐OES) on samples collected from the historical quarries. Normative mineral weight percentages were calculated from the chemical data using the CIPW norm (Cross, Iddings, Pirsson, and Washington). Because hydrated mineral phases are not considered in this calculation, H₂O was excluded. The resulting normative values were subsequently plotted in the Q′–ANOR′ diagram.

The three varieties of granites studied were cut in cubic samples (6×6×6 cm) and subjected to 90 cycles of the following accelerated ageing treatments in a simulation chamber under controlled conditions:

- T1: Freezing/thawing and cooling/heating (−20 to 110 °C)^48^. After drying at 60°C to constant weight, the samples were fully immersed in distilled water for 16 hours, then cooled and maintained at – 20 °C for 3 hours. The temperature was subsequently raised to 110 °C and held for an additional 3 hours. Following cooling to laboratory temperature for 2 hours, the entire process was repeated.
- T2: Sulphate crystallisation (Na_2_SO_4_ • 10H_2_O)^46^. After drying at 60 °C to constant weight, each sample was immersed in a 14% solution of hydrated sodium sulphate (Na_2_SO_4_ • 10H_2_O) for 2 hours, with the container sealed to preserve concentration. The samples were then collected from the container and allowed to drain at room temperature for 15 minutes. Subsequently, they were placed in an oven at 105 °C for 16 hours, with high relative humidity maintained by placing a porcelain container filled with water inside the oven. Finally, the samples were collected and placed in a desiccator for 5 hours and 45 minutes before the next cycle began.
- T3: The experiment was carried out over a combined freezing/thawing and cooling/heating treatment with sulphate crystallisation. We used the method T1, but the samples were immersed in a 14% solution of hydrated sodium sulphate (Na_2_SO_4_•10H_2_O) instead of distilled water^48^.

The ultrasound propagation speed was measured with an ULTRASONIC Tester BP-5 from STEINKAMP during cycles 0 (before starting each of the artificial ageing treatments), 30, 60, and 90. This property was determined on a total of 27 specimens, that is, three repetitions for each of the three granite types and three types of ageing, with measurements taken at cycles 0, 30, 60, and 90 of the different accelerated artificial ageing processes.

To study the compressive strength (mechanical property), a linear regression model^21^ was used, with compressive strengh (ECR; which is the maximum load per unit area that is capable of supporting a specimen up to failure) as the dependent variable and mean ultrasound propagation speed as independent variable [V=(Vx+Vy+Vz)/3]. A dummy variable named “altered” (0=not altered and 1=altered) was included. The coefficient of determination was R^2^ = 0.586. The linear regression model was:

$$ECR \, = \, 146.983 \, + \, 0.203 \, x \, V \, {-} \, 153.971 \, Altered$$

Where:

ECR Estimated Compressive Strength (Kg/cm^2^)

V Mean ultrasound propagation speed (m/s)

Those compressive strength values are estimates and that the difference between these values and those that could have been obtained experimentally through the corresponding test depends, among other factors, on the quality of the fit. This was achieved, in part, through the experimental determination of compressive strength on granite samples subjected to 90 cycles of each of the accelerated artificial ageing tests considered in this study, in accordance with UNE-EN 1926.^49^ testing standard. The test was performed on three cubic specimens with an edge length of 60 mm. The loading faces were flat, and the lateral faces of the specimens were smooth, free of irregularities, and rectilinear. Prior to testing, the specimens were dried at 70 ± 5 °C until constant mass was achieved. Subsequently, they were stored at 20 ± 5 °C until thermal equilibrium was reached, and the test was then performed.

A uniformly distributed load was applied and continuously increased until failure occurred. The uniaxial compressive strength (MPa) was calculated using the following equation:

$$CR \, = \, F/A$$

where *F* is the failure load (N) and *A* is the cross-sectional area of the specimen prior to testing (mm^2^).

The experimental compressive strength values were originally determined in MPa, as indicated in the text, in accordance with the standard. However, in Table 1, these values were converted to kg/cm^2^ in order to allow direct comparison with the compressive strength values estimated from the linear regression model, which are expressed in these units.

**Table 1:** Average estimates of resistance to compression from the regression model^21^ (ECR) and average values experimentally determined for the three granites after 90 cycles of each of the artificial accelerating ageing tests (CR).

Sample	Ageing	Cycles	ECR*	CR*
C	No	0	1019	
F	No	0	922	
G	No	0	841	
C	T1	30	858	
F	T1	30	766	
G	T1	30	652	
C	T2	30	828	
F	T2	30	744	
G	T2	30	648	
C	T3	30	777	
F	T3	30	621	
G	T3	30	558	
C	T1	60	850	
F	T1	60	755	
G	T1	60	629	
C	T2	60	805	
F	T2	60	719	
G	T2	60	604	
C	T3	60	696	
F	T3	60	544	
G	T3	60	275	
C	T1	90	826	925
F	T1	90	732	908
G	T1	90	603	847
C	T2	90	782	776
F	T2	90	698	721
G	T2	90	573	637
C	T3	90	422	549
F	T3	90	248	457
G	T3	90	140	107

^*^Kg/cm^2^

The test was conducted using a 300-ton compression testing machine (CME-300/SDC, Sistema de Ensayos S.L.), belonging to INTROMAC (Technological Institute of Dimension Stone and Construction Materials of the Regional Government of Extremadura, Cáceres, Spain).

A three-way mixed MANOVA was used to study the influence in the quarry samples (Ávila Grey Granite [C], Airón Aplitic Granite [F] and Airón Biotitic Granite [G]) of the number of ageing cycles (30, 60, and 90) and the different ageing treatments (T1: freezing/thawing and cooling/heating, T2: salt crystallisation, and T3: freezing/thawing and cooling/heating + salt crystallisation). This MANOVA considered two factors, between-subjects (granite variety and ageing treatment) and within-subjects factors (ageing cycle), and four dependent variables (Δm, ΔVx, ΔVy, and ΔVz). A *p-*value < 0.05 was considered statistically significant. Due to the multivariate nature of the study and the multiple comparisons, the family-wise error rate would be increased. To account for this error, the Bonferroni adjustment method was used.

## Results and discussion

**Fig. 4:**
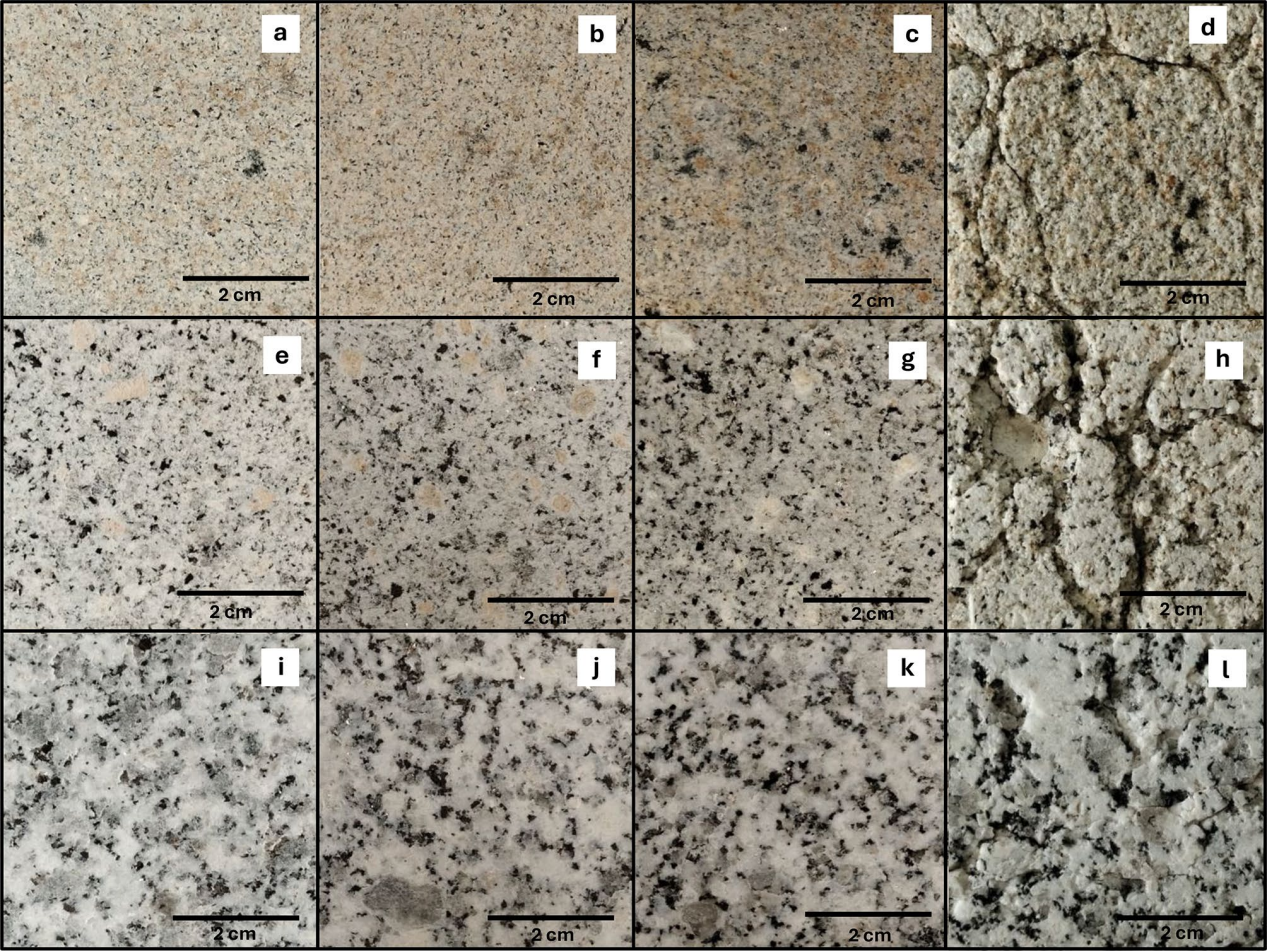
The appearance of the quarry samples: (**a**) F, (**e**) G and (**i**) C, and of the samples subjected up to cycle 90, for each of the accelerated artificial ageing tests T1, T2 and T3 and each of the materials studied (F, G, and C): (**b**) F, T1; (**c**) F, T2; (**d**) F, T3; (**f**) G, T1; (**g**) G, T2; (**h**) G, T3; (**j**) C, T1; (**k**) C, T2 and (**l**) C, T3.

The appearance of the quarry samples and of the samples subjected up to cycle 90, for each of the accelerated artificial ageing tests and each of the materials studied (F, G, and C), are shown in Fig. 4, in which it can be observed that ageing processes T1 and T2 are less aggressive than T3. T2 produces greater damage than T1, although this is practically imperceptible to the naked eye. At T3, the Airón granites (varieties F and G) show pronounced weathering, especially associated with the development of microcracking, while the Grey Ávila Granite (variety C) displays markedly lower deterioration, largely restricted to superficial alterations. All of this is a result of the stresses caused by differential thermal and structural expansions and contractions of minerals that are more easily degradable, such as biotites and feldspars^50^. When these stresses exceed the elastic limit, they produce microcracks.

The results derived from the three-way mixed MANOVA analysis indicate a different behaviour resulting from the controlled cycles (30, 60, and 90) for the different types of ageing. The interaction granite type*ageing type was not significant for cycle 30, whereas a significant interaction for cycles 60 and 90 was found. Therefore, in some cases, the Main effects (overall influence of one factor, regardless of the other) were studied, whereas in other cases the Simple Main effects (influence of one factor at specific levels of the other factor) were analysed.

### Artificial ageing treatment

**Fig. 5:**
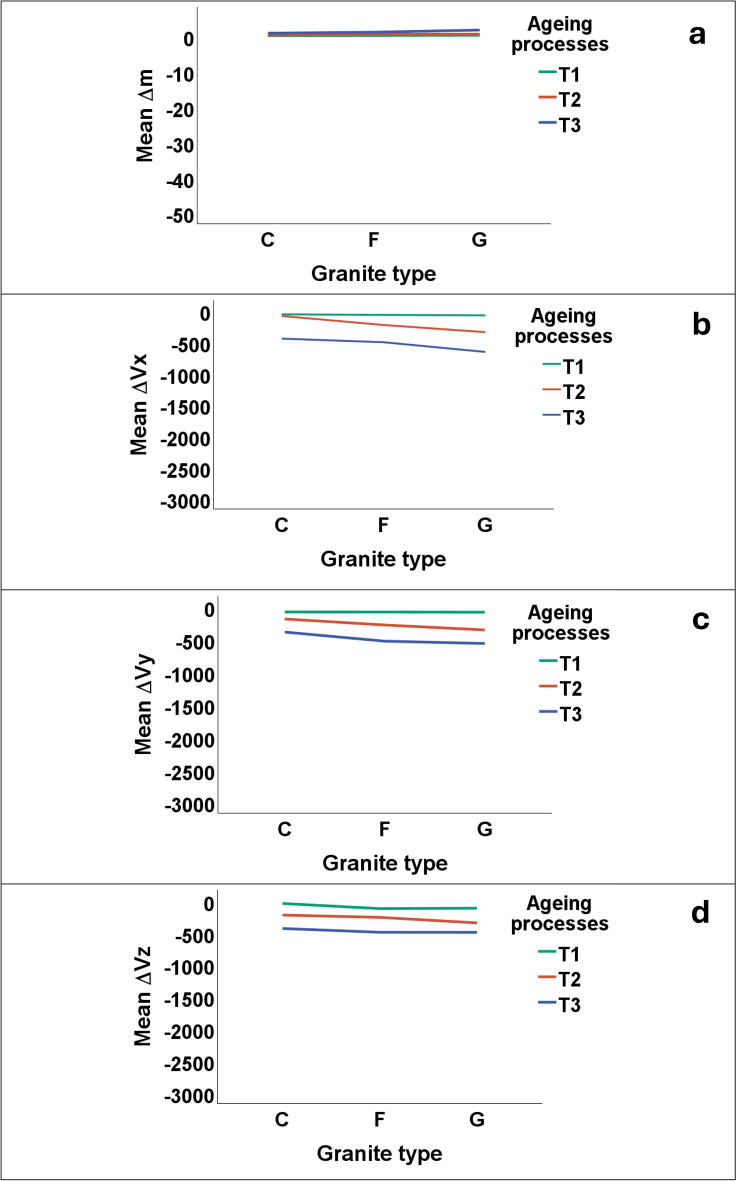
For all granite types, each ageing test, and the controlled cycle 30, this figure shows the mean values of: (**a**) Δm, (**b**) ΔVx, (**c**) ΔVy and (**d**) ΔVz.

**Fig. 6:**
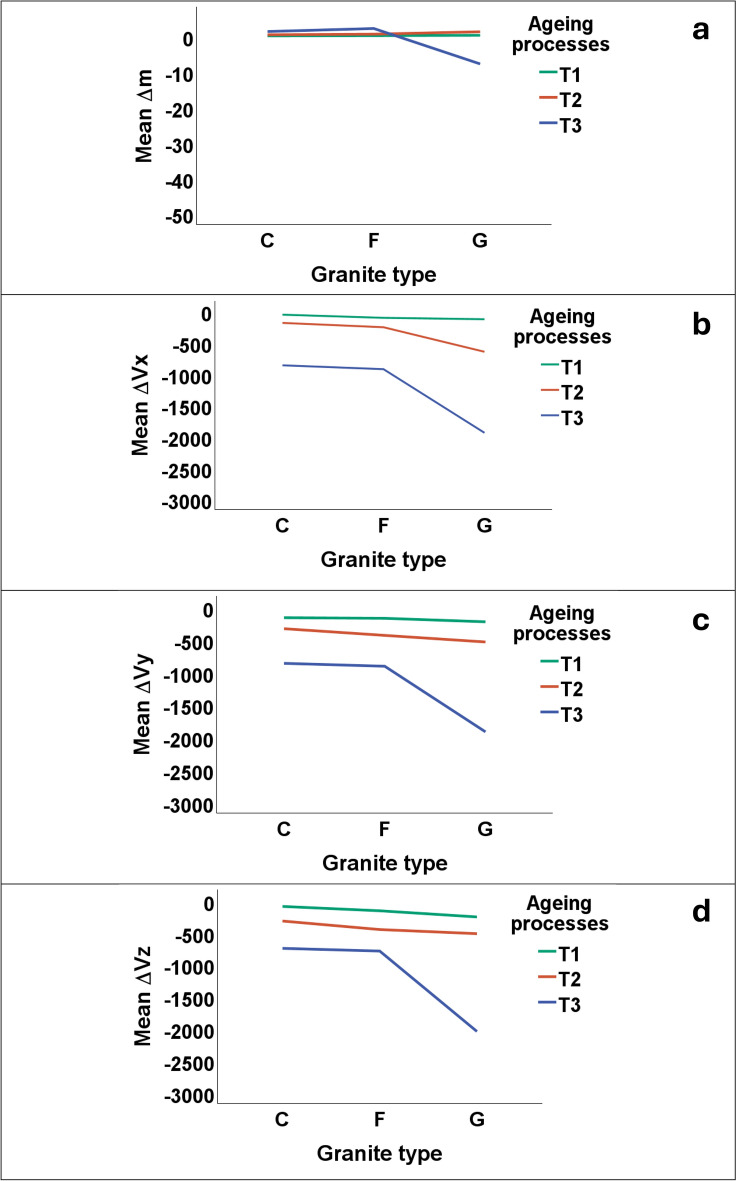
For all granite types, each ageing test, and the controlled cycle 60, this figure shows the mean values of: (**a**) Δm, (**b**) ΔVx, (**c**) ΔVy and (**d**) ΔVz.

**Fig. 7:**
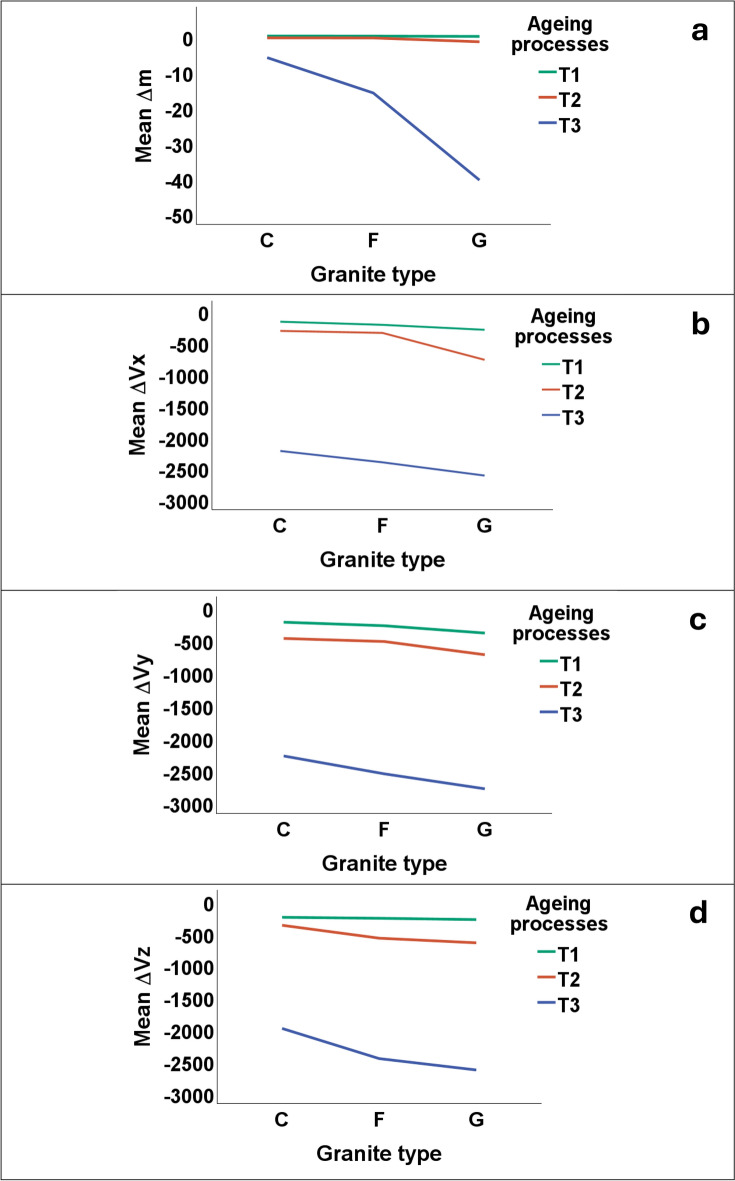
For all granite types, each ageing test, and the controlled cycle 90, this figure shows the mean values of: (**a**) Δm, (**b**) ΔVx, (**c**) ΔVy and (**d**) ΔVz.

During the first 30 cycles, all three types of ageing caused an increase in mass (Fig. 5a), regardless of granite variety. This increase was greater for the T3 process, indicating that the porosity (already present or created during these first 30 cycles) of the different granites was being filled with the salt necessary to carry out the ageing process. The slight weight gain observed for the T1 ageing could be due to the dragging of clays because of this process, which could have caused a clogging of the pore network and left part of the water retained in the sample. This increase in mass was maintained by the end of cycle 60 (Fig. 6a), except for T3 ageing, which caused a decrease in the G granite variety. Significant differences in Δm were found in the granitic variety G between the T3 process compared to T1 and T2, suggesting that T3 ageing is the most aggressive and the granitic variety G is the most alterable of those studied. After 90 cycles, the degradation pattern induced by T3 had led to mass loss in all granite types. (Fig. 7a). Significant differences were observed between T3 and treatments T1 and T2.

The values of ΔVx, ΔVy, and ΔVz revealed a progressive degradation characterised by a strong interaction between ageing processes and granite type. During the initial stage (30 cycles), deterioration was moderate (Fig. 5b-d); while treatment T1 remained inert, the T3 process induced the first significant velocity losses, prematurely establishing the vulnerability of granite G. From this early stage, the hierarchy of treatment impact was clearly defined (T3 > T2 > T1) and consistently replicated across ultrasound propagation speeds. However, the critical turning point occurred at 60 cycles (Fig.6b-d): the interaction intensified drastically under T3, causing a strong drop in velocity for granite G. While varieties C and F maintained moderate and stable degradation. Upon completion of the 90 cycles, the three variables converged into a scenario of severe and generalized degradation (Fig. 7b-d), dominated by the aggressiveness of the T3 treatment. Meanwhile, treatment T2 maintained a selective deterioration profile (primarily affecting granite G), whereas T1 confirmed its long-term inertness, registering no significant variations in any of the studied variables.

In addition, the alteration produced by the accelerated artificial ageing used in all granitic varieties would be, in descending order, the following:

$$T3 \, > \, T2 \, > \, T1$$

These findings have already been confirmed by the results obtained on these same granites that determine other physical properties such as colour variations^46^, and other granites from the regions of Ávila and Segovia, although in the latter case, only concerning the relationship between T1 and T3^21^. In addition, similar conclusions have been reached, either fully or partially, regarding other types of stone materials when comparing two types of ageing treatments; for instance, this has been observed in conglomerates and sandstones from Zamora^45^, Villamayor sandstone^13^, andesites^24^, sandstones^22^, etc. Studies carried out on granites other than those analysed in this work show that they are similarly affected or deteriorated, whereas other materials are much less affected.

### Granite type

**Fig. 8:**
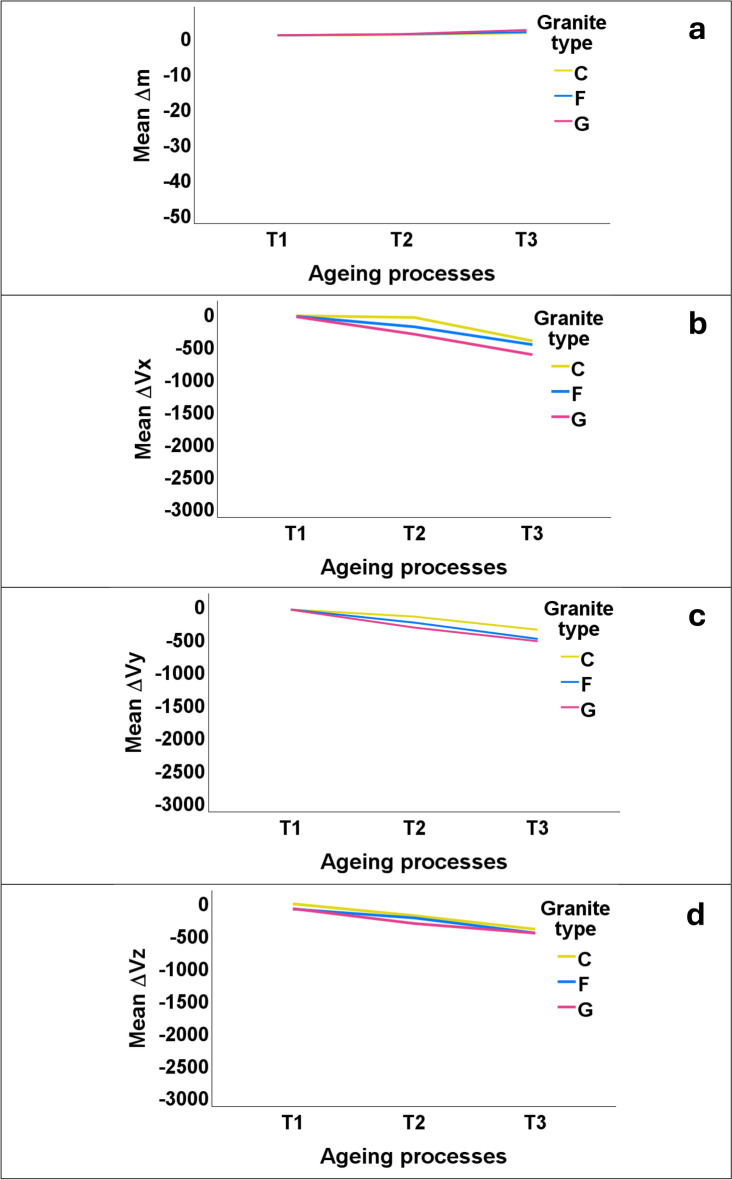
For all ageing test, each granite type, and the controlled cycle 30, this figure shows the mean values of: (**a**) Δm, (**b**) ΔVx, (**c**) ΔVy and (**d**) ΔVz.

**Fig. 9:**
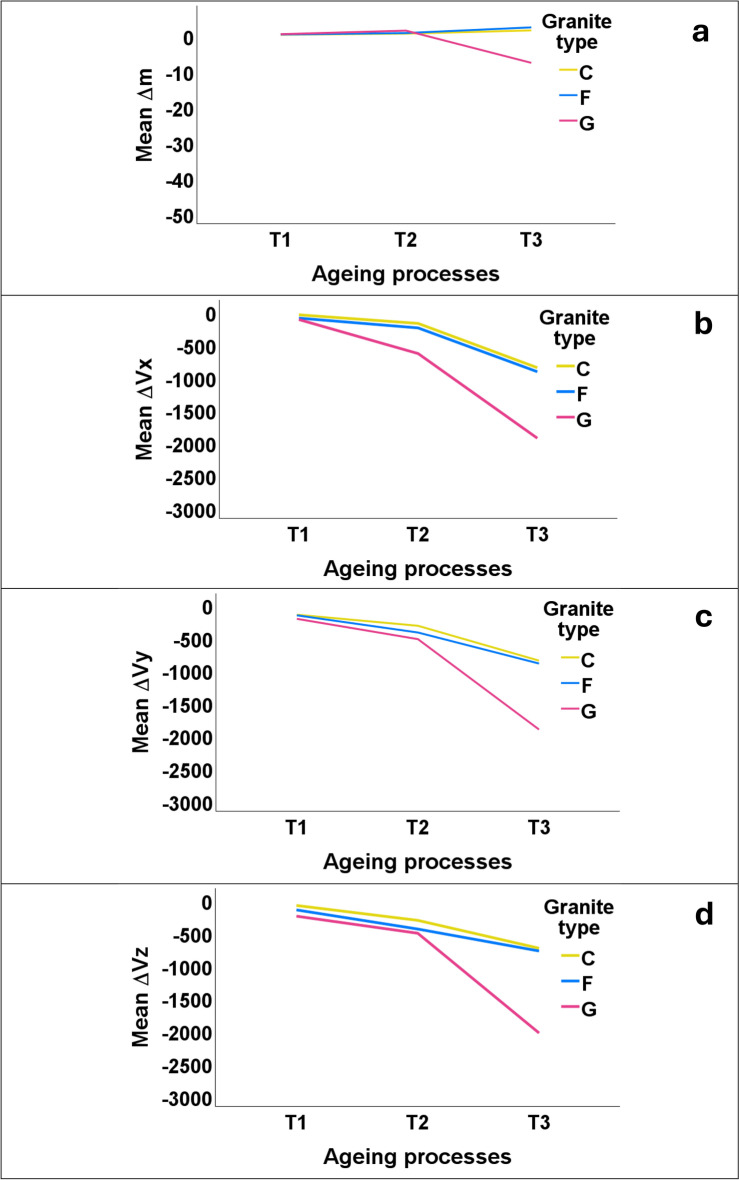
For all ageing test, each granite type, and the controlled cycle 60, this figure shows the mean values of: (**a**) Δm, (**b**) ΔVx, (**c**) ΔVy and (**d**) ΔVz.

**Fig. 10:**
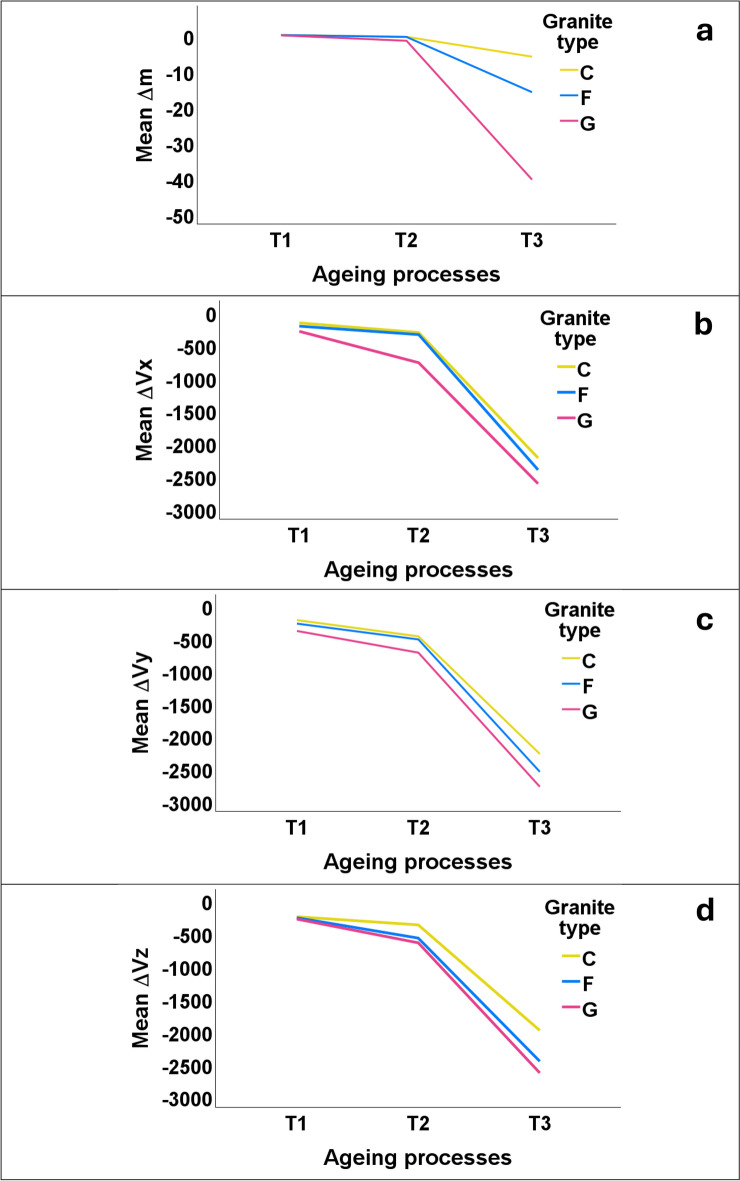
For all ageing test, each granite type, and the controlled cycle 90, this figure shows the mean values of: (**a**) Δm, (**b**) ΔVx, (**c**) ΔVy and (**d**) ΔVz.

The temporal evolution of Δm revealed a progressive pattern of lithological discrimination. During the first 30 cycles, there are no discernible differences among the granite varieties (Fig. 8a). By 60 cycles, the onset of divergence was detected, identifying exclusively granite G as vulnerable to treatment T3 (Fig. 9a). Finally, at 90 cycles (Fig. 10a), the data evidenced a significant divergence in material response under treatment T3, establishing a clearly stratified resistance gradient (C > F>>G). While granite C maintained notable stability, granite G underwent a drastic reduction. This quantitative distinction confirms the high sensitivity of this variable in discriminating lithological susceptibility to accelerated ageing.

At 30 cycles, while ΔVx, ΔVy, and ΔVz exhibited similar visual trends (Fig. 8b-d). Significant differences were detected between granites C and G under treatments T2 and T3. Upon reaching 60 cycles (Fig. 9b-d), a diagnostic convergence occurred under the most aggressive scenario (T3) and the treatment T2. Statistical analysis confirmed that, for all monitored variables, granite G suffered a significant decay, clearly distinguishing itself from the behaviour of varieties C and F. This indicates that by 60 cycles, a critical resistance threshold has been surpassed for lithology G. At the conclusion of the 90 cycles, all variables exhibited steep declines (Fig. 10b-d), suggesting a common decay. Notably, under the most aggressive scenario (T3), significantly distinguishing granite C not only from G but also from F, thereby consolidating variety C as the most durable under extreme ageing conditions.

Based on the results obtained, it can be observed that the order in which the granitic varieties were affected by the alteration resulting from the accelerated artificial ageing is as follows:

$$Variety \, G \, > \, Variety \, F \, > \, Variety \, C$$

Extensive microcracking was identified in the so-called Ávila Grey granite (C), with intra-, inter-, and trans-mineral cracks frequently filled with salts^51^. Feldspars and plagioclases were heavily fractured, while quartz grains were fragmented and embedded in saline masses. Small biotite crystals showed openings along exfoliation planes, with salts precipitating in the spaces thus created. The deterioration was attributed to the synergistic effects of physical or mechanical weathering processes, including thermoclasty, gelifraction, and salt crystallisation (haloclasty). Similar findings were obtained through controlled ageing experiments on granites from Segovia^21^, where changes in colour and ultrasonic propagation speed were correlated with different ageing cycles (T1 and T3), demonstrating that decrease in ultrasonic propagation speed, can serve as a quantitative indicator of microstructural damage.

Furthermore, Fort et al.^52^ analysed granites used in historical constructions in the region of Madrid, showing that salt crystallisation and freeze/thaw cycles exert a significant influence on ultrasonic and physical properties. The authors highlighted that variations in microclimate and environmental exposure strongly affect the decay rate of building stones. Granites from Madrid^52^ and Segovia^21^, although mineralogically similar to those from Ávila^46^, showed slightly greater decreases in ultrasonic speed under equivalent artificial ageing, suggesting differences in porosity and crack connectivity.

### Durability of granites

Table 1 shows the predicted mean compressive strength (ECR) values obtained from the regression model for all sample types at cycles 0, 30, 60 and 90. Additionally, the experimentally determined mean values of compressive strength are presented for the three granite lithofacies following 90 cycles of each artificial accelerated ageing test. All laboratory experimental results of this workhave been published in the Digital.CSIC repository^53^.

Model validation was performed by comparing experimentally determined values with out-of-sample predictions for cycle 90. Predictive performance was evaluated using the root mean squared error (RMSE = 131.15), mean absolute error (MAE = 108.46), and mean absolute percentage error (MAPE = 22.94%), indicating satisfactory predictive accuracy. All numerical computations were performed using base functions (R version 4.5.1).

According to the durability shown by the different granites after the application of the treatments, the accelerated artificial ageing tests can be classified, in descending order, as follows:

$$T1 \, > \, T2 \, > \, T3$$

This decrease in durability is much higher for T3 than for T2 and T1 in all the different varieties studied. This could be explained by the synergistic action of the two processes involved in T3, which enhance the degradation and increase the kinetics of this process compared to T1 and T2 independently^7^.

## Conclusions

The main conclusions of this work are the following:

- The use of the three-way mixed MANOVA statistical technique has allowed us to determine the level of impact on each of the granitic varieties studied with the different accelerated artificial ageing processes used in this study. Subsequently, the determination of the estimated compressive strength has allowed us to confirm the coherence of the findings derived from the statistical analysis and to determine the durability of each of the granitic varieties under the different accelerated artificial ageing processes studied.
- The order of the different granitic varieties according to their durability, as determined by MANOVA and confirmed by the estimated compressive strength, as follows:
  $$Variety \, C \, > \, Variety \, F \, > \, Variety \, G$$
  c) Artificial ageing T3 is the most aggressive of the three ageing processes tested due to the synergic action of two processes (freezing/thawing and cooling/heating [T1], and salt crystallisation [T2]), which increases the kinetics of the degradation processes, with the greatest variations for all the variables studied between the granites subjected to the ageing process and the corresponding quarry granites. However, these variations are smaller for the other two ageing processes, which correspond to the two separate processes (T1 and T2).
- The variety most resistant to (experimental) physical weathering processes produced by gelifraction and thermoclasty (T1), haloclasty (salt crystallisation by sulphates, T2), and their synergistic effects (haloclasty + gelifraction + thermoclasty, T3) is the Ávila Grey granite; this is possibly due to the late‑magmatic recrystallisation associated with its genesis, which seals microcracking. The least resistant variety is the biotitic Airón granite, as a result of prior alteration (in the quarry) that affected the plagioclases (albite/anorthite).
- The damage caused by the different accelerated artificial ageing processes studied across various granite varieties depends on their internal characteristics, which are primarily mineralogical. This is due to stresses arising from differential thermal and structural expansions and contractions of minerals that are more easily degradable, such as biotites and feldspars. When such stresses exceed the elastic limit, they produce microcracks.

## Data Availability

All laboratory experimental results of this work have been published in the Digital.CSIC repository49
